# Rapid and Low Cost Manufacturing of Cuff Electrodes

**DOI:** 10.3389/fnins.2021.628778

**Published:** 2021-02-16

**Authors:** Matthew T. Flavin, Marek A. Paul, Alexander S. Lim, Senan Abdulhamed, Charles A. Lissandrello, Robert Ajemian, Samuel J. Lin, Jongyoon Han

**Affiliations:** ^1^Department of Electrical Engineering and Computer Science, Massachusetts Institute of Technology, Cambridge, MA, United States; ^2^The Charles Stark Draper Laboratory, Inc., Cambridge, MA, United States; ^3^Division of Plastic and Reconstructive Surgery, Department of Surgery, Beth Israel Deaconess Medical Center, Harvard Medical School, Boston, MA, United States; ^4^Department of Neurosurgery, Lower Silesia Specialist Hospital, Wrocław, Poland; ^5^McGovern Institute for Brain Research, Massachusetts Institute of Technology, Cambridge, MA, United States; ^6^Department of Biological Engineering, Massachusetts Institute of Technology, Cambridge, MA, United States

**Keywords:** multipolar, flexible electronics, rapid prototyping, subtractive manufacturing, multichannel, neuro-modulation, circumferential, functional electrical stimulation (FES)

## Abstract

For many peripheral neuro-modulation applications, the cuff electrode has become a preferred technology for delivering electrical current into targeted volumes of tissue. While basic cuffs with low spatial selectivity, having longitudinally arranged contacts, can be produced from relatively straightforward processes, the fabrication of more complex electrode configurations typically requires iterative design and clean-room fabrication with skilled technicians. Although facile methods for fabricating cuff electrodes exist, their inconsistent products have limited their adoption for rapid manufacturing. In this article, we report a fast, low-cost fabrication process for patterning of electrode contacts in an implantable peripheral nerve cuff. Using a laser cutter as we have prescribed, the designer can render precise contact geometries that are consistent between batches. This method is enabled by the use of silicone/carbon black (CB) composite electrodes, which integrate with the patterned surface of its substrate—tubular silicone insulation. The size and features of its products can be adapted to fit a wide range of nerve diameters and applications. In this study, we specifically documented the manufacturing and evaluation of circumpolar cuffs with radial arrays of three contacts for acute implantation on the rat sciatic nerve. As part of this method, we also detail protocols for verification—electrochemical characterization—and validation—electrophysiological evaluation—of implantable cuff electrodes. Applied to our circumpolar cuff electrode, we report favorable electrical characteristics. In addition, we report that it reproduces expected electrophysiological behaviors described in prior literature. No specialized equipment or fabrication experience was required in our production, and we encountered negligible costs relative to commercially available solutions. Since, as we demonstrate, this process generates consistent and precise electrode geometries, we propose that it has strong merits for use in rapid manufacturing.

## Introduction

Whether manufactured in-house or purchased commercially, the associated costs and lead-times for implantable electrical devices will be prohibitive to many researchers. This limitation motivated the work we present in this article: a rapid manufacturing method for minimizing the time and cost associated with designing and producing new cuff electrodes for peripheral nervous system (PNS) stimulation. Our goal is to enable researchers to quickly build and improve prototypes for cuff electrodes, controlling parameters such as electrode sizes and locations. Furthermore, our goal is to enable this for the broadest group of researchers as possible in terms of their fabrication experience and available equipment.

Over the last decade, several targets in the PNS have emerged as potential substrates for therapeutic intervention. Direct electrical modulation of the vagus nerve has been approved by the United States Food and Drug Administration (FDA) for the treatment of drug-resistant epilepsy (Englot et al., 2011) and major depressive disorder (Berry et al., 2013), and investigators are exploring other potential indications such as chronic pain (Multon and Schoenen, 2005), cardiac arrhythmia (Brack et al., 2013), and autoimmune disorders (Koopman et al., 2017). Electrical stimulation of peripheral nerves can also help patients with neurological damage restore sensation and control of muscles (Navarro et al., 2005). This modality, a subset of functional electrical stimulation (FES), has been successfully applied in therapeutic devices such as ActiGait, an implanted peripheral nerve stimulator for which Neurodan A/S won the CE mark (Conformité Européene) to treat foot drop in patients with stroke (Burridge et al., 2007).

Current peripheral neuro-modulation approaches typically involve either intraneural or cuff electrode formats. The latter confers several advantages—the characteristically larger geometry of cuff electrodes eases some surgical constraints and permits the incorporation of contacts with larger surface areas. As a result, the device can deliver larger currents without incurring unintended redox processes, capable of damaging both the electrode and the surrounding tissue (Cogan, 2008). In addition, cuff electrodes can be implanted without directly cutting or penetrating the nerve—a technically challenging procedure that is prone to acute and long-term tissue damage (Navarro et al., 2005).

**FIGURE 1:**
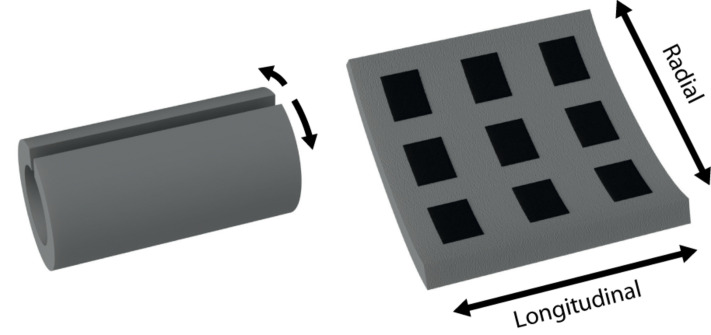
Illustration of longitudinal and radial arrangements of cuff electrode contacts.

While basic cuff electrodes with longitudinally arranged contacts can be produced from relatively straightforward processes (Foldes et al., 2011), the fabrication of more complex electrode configurations typically requires laborious design and clean-room fabrication with skilled technicians. The circumpolar cuff electrode, with contacts arrayed radially around the cuff’s interior, is one such configuration (see Figure 1 for an illustration of radial versus longitudinal arrangements). In circumstances where sophisticated fabrication methods are feasible, thin-film processes such as that described by Navarro et al. (2001) for spiral cuffs as well as that for the recently introduced “split-ring” electrode can produce circumpolar electrodes with finely structured geometries (Tsang et al., 2010; Lee et al., 2017; Cobo et al., 2019).

The utility of circumpolar electrodes is that they can selectively target groups of fascicles within a nerve bundle by directing stimuli into adjacent contacts (Sweeney et al., 1990; Rozman et al., 1993; Veraart et al., 1993; Goodall et al., 1996; GrillJr., and Mortimer, 1996; Navarro et al., 2001; Tarler and Mortimer, 2004). Applied to FES, Polasek et al. (2009) demonstrated in a clinical trial that a circumpolar cuff electrode could reliably activate single muscles, applying the effects of stimulation discriminatorily. Likewise, spatial selectivity has significant advantages in neuro-modulation modalities such as vagus nerve stimulation—as discussed by Aristovich et al. (2019), the use of a cuff electrode with radially fragmented contacts limits off-target stimulation and reduces adverse effects.

The elaborate nature of the multilayer thin-film processes, however, makes them untenable for rapid manufacturing of cuff electrodes. The demand for rapid manufacturing processes has increased dramatically over the last decade with the introduction of tools such as 3–D printers and laser cutters. This growth has been driven by three crucial use cases: (1) manufacturing products with low, sporadic demand, (2) design-stage production for quick iteration of ideas, and (3) manufacturing customizable products (Karapatis et al., 1998; Goole and Amighi, 2016). Customization is particularly valuable for biomedical applications, for which product requirements can vary substantially between subjects. Investigators are currently evaluating rapid manufacturing methods to custom fit prosthetic limbs (ten Kate et al., 2017) and fabricate tissue scaffolds (Do et al., 2015).

Each of the use cases listed above highlight the importance of an effective rapid manufacturing method for cuff electrodes. Regarding (1), due to the large parameter space for designing cuff electrodes, particular configurations have sporadic demand. Since this prevents commercial manufacturers from enjoying economy of scale, they might benefit from adopting a rapid manufacturing solution. This translates into lower costs for academic laboratories in particular, which typically purchase in low volumes. Regarding (2), rapid iteration of designs would reduce time and costs associated with cuff electrode development, particularly in early stages. For small businesses and startups that want to avoid contracting out this work, rapid manufacturing would provide an alternative to ramping up expensive facilities. Finally, regarding (3), this approach could allow technicians to manufacture cuff electrodes on demand to meet the individual requirements of each subject or patient. A patient with post-amputation pain, for example, would benefit from being fitted with a custom sized implant based on the diameter of their sciatic nerve, which can vary between 1.4 and 1.9 mm in the Gluteal region (Rai, 2015). Aside from Tong et al. (2018) who describe the use of robotic stitching to embed platinum wires in silicone, no technology currently exists that serves this application. Similar to the clinical setting, academic laboratories have diverse requirements and would benefit from producing cuff electrodes in-house using a rapid manufacturing process.

Despite current production challenges, several commercial solutions for cuff electrodes with radially arranged contacts exist (see Table 1 for a comparison). However, the associated costs reflect those production challenges, requiring, even for pilot studies, a significant investment.

**TABLE 1:** Comparison of commercial cuff electrodes with radially arranged contacts.

Supplier	Configurations*	Substrate/electrode material*	Method	Related References
Ardiem Medical, Inc.	1–6 radial 1–6 longitudinal	Silicone/Pt	Pt foil	Foldes et al., 2011; Haugland, 1996; Naples et al., 1988; Rozman et al., 2018; Veraart et al., 1993
World Precision Instruments, Inc.	2–8 radial 1–9 longitudinal	Silicone/Pt, PtIr, stainless-steel	Manual stitching	Loeb and Peck, 1996; Rios et al., 2019
Microprobes for Life Science	2–8 radial 1–9 longitudinal	Silicone/Pt, PtIr, stainless-steel	Manual stitching	Loeb and Peck, 1996; Rios et al., 2019
CorTec, GmbH	Not advertised	Silicone/PtIr	Laser-etched metal foil	Ordonez et al., 2014

*Based on publicly advertised products.

Meanwhile, for in-house production of cuff electrodes, few processes exist that are rapid and economical, and even fewer broadly accessible in terms of their requirements for specialized equipment and technical skill. Naples et al. (1988), Haugland (1996), and Foldes et al. (2011) demonstrate facile methods using platinum foil, but these are not feasible for complex configurations such as circumpolar electrode designs without a high level of technical skill and manual dexterity (Veraart et al., 1993; Dweiri et al., 2016; Rozman et al., 2018). Furthermore, since they require that contacts be patterned by hand, the consistency of the electrode geometry will vary significantly with the skill of the operator. More recently, investigators such as Stieglitz et al. have shown that these issues can be resolved by employing a laser cutter to pattern the platinum foil; however, their method requires multiple alignment and mask etching steps which significantly increase the technical skill and equipment required, along with time and cost (Ordonez et al., 2014). Loeb and Peck (1996) report a method of fabricating cuffs by stitching platinum wires, which, as a facile process, is more amenable to circumpolar arrays than embedding platinum foil (Rios et al., 2019). However, as stated by Haugland (1996), “With this design it is difficult to control the exact position and size of the electrodes, and lose [sic] wires may act as constrictions of the nerve.” In addition, Hoffer and Kallesoe (2000) report that hand sewn wires pose a risk of cutting into the nerve due to misalignment and splaying. Inadvertent piercing of the nerve is also a risk for some of the foil electrode processes that require welding or soldering of lead wires (particularly for interfaces of dissimilar metals, such as platinum–stainless steel), as mechanical weak points can arise at the joints (Hoffer and Kallesoe, 2000).

In this article, we report an alternative solution: silicone/carbon black (CB) composite electrodes embedded in tubular silicone insulation. Their sizes and features can be adapted to fit a wide range of nerve diameters and applications. A batch of four costs approximately $8.80 of materials and three to four hours of labor. Furthermore, it requires no specialized equipment or fabrication experience. While this method requires some manual assembly to attach lead wires, it permits the use of a laser cutter to pattern the electrode contacts. Therefore, unlike Naples et al. (1988), Haugland (1996), and Foldes et al. (2011) who require manual assembly in all steps, this method will produce consistent electrode geometries with a high precision relative to the dimensions of the nerve.

We selected our electrode material, a CB composite (Reyes et al., 2014; Eklund and Kjäll, 2019), so that it would integrate seamlessly with the silicone body of the cuff. Graphitized carbon has similar characteristics to CB, but CB was chosen since its composite with silicone has more favorable bulk conductivity (Quinsaat et al., 2019). Several other silicone composites, such as those of platinum powder (Minev et al., 2015; Schiavone et al., 2018) and carbon nanotubes (CNTs) (Behrens et al., 2018; Kim et al., 2018), demonstrate better charge transfer impedances. However, CB is far cheaper and easy to disperse. Carbon-based powders such as graphite and CNTs have demonstrated a cytotoxic effect on cell viability at a high enough concentration (Fiorito et al., 2006), but polymer composites of these materials show long-term biological inertness (Blau et al., 2011; Kim et al., 2018). The silicone/graphite composite electrode from Blau et al. (2011), for example, was incubated with a culture of cortico–hippocampal neurons and demonstrated healthy morphological characteristics for over three weeks *in vitro*.

Using our process, we manufactured 3-channel circumpolar nerve cuffs for implantations on rat sciatic nerves. Note that, as stated by Navarro et al., the selectivity of fascicular activation generally increases with the number of embedded contacts (Navarro et al., 2005). Considering this, one would likely improve outcomes by implementing a larger array.

We subjected our 3-channel devices to both verification via electrochemical characterization and validation via an electrophysiological study. This electrophysiological study entailed the acute implantation of a circumpolar cuff onto the sciatic nerve of an anesthetized rat, and the measurement of compound muscle action potentials during excitatory stimulation. Although the significance of this particular animal study is limited by its small sample size, we include it here to highlight appropriate methodological steps for validating cuff electrodes produced under our process. With this in mind, its results reproduce key electrophysiological behaviors, including fascicle selectivity, reported in previous literature, and this further supports the quality of our process (Sweeney et al., 1990; Rozman et al., 1993; Veraart et al., 1993; Goodall et al., 1996; GrillJr., and Mortimer, 1996; Navarro et al., 2001; Tarler and Mortimer, 2004).

## Materials and Methods

This section, in addition to detailing the steps for fabricating a 3-channel circumpolar cuff electrode, includes instructions for design verification and validation. In this case, verification entails electrochemical methods for evaluating characteristics such as impedance and charge injection capacity. Validation entails the execution of a motor unit recruitment study on the sciatic nerve of an anesthetized rat.

### Device Fabrication

**FIGURE 2:**
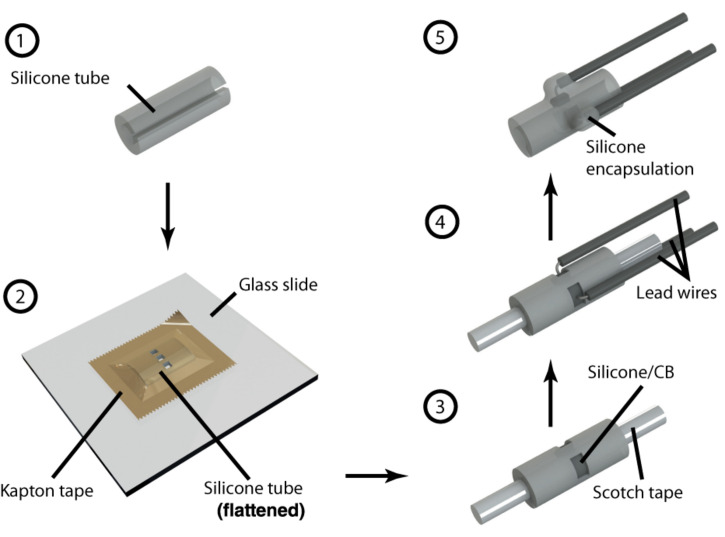
Step by step illustration of cuff electrode fabrication. Each number corresponds to a step in the fabrication process as described in the “Device Fabrication” section. References in this paper to Contacts 1, 2, and 3 each correspond to the three depicted here.

We fabricated our cuff electrode in five steps, depicted in Figure 2 and described below:

*Step 1.* The process starts with preparing the substrate, an 8 mm section of silicone tubing (Versilon SPX-50), by opening it with a longitudinal cut. We used tubing with a 1.6 mm inner diameter and 3.2 mm outer diameter to suit our target, 2 cm of exposed nerve with a diameter in the range 1–1.5 mm. Although we focused exclusively on nerves of this size in our experiments, smaller nerve diameters could be accommodated by starting with narrower tubing—the only limiting factor is the required electrode size, of which, as discussed in the following steps, the laser resolution and wire assembly are factors. At the time of writing, narrower tubing with appropriate characteristics can be purchased from suppliers such as Trelleborg.

*Step 2.* To pattern the contacts, we flattened the tubing inside-down against a glass slide using a piece of polyimide (PI) tape with silicone adhesive (1 Mil Kapton tape from Dupont). Using a computer numerical control (CNC) laser cutter (PLS4.75 from Universal Laser Systems), we then aligned and cut channels through the substrate according to a drawing of our intended electrode configuration. For this study, we chose to pattern a radial array of three rectangular contacts, each 0.9 × 1.4 mm^2^, spaced 0.6 mm apart. These electrodes could be made as small as the resolution of the laser permitted, which, in our case, was ∼200 μm. As such, precision could be improved in this step by calibrating the laser intensity to the minimum capable of cutting through both Kapton tape and silicone tubing. Afterwards, we released the patterned substrate from the glass slide and cleaned off any residue left by the laser using isopropyl alcohol and cotton-tipped applicators.

*Step 3.* The patterned substrate was then laminated onto a cylindrical scaffold composed of a metal wire wrapped with transparent pressure-sensitive tape (Scotch tape from 3M; adhesive side facing out). Products such as Kapton tape with silicone-based adhesives must be avoided for this step, as the strong bond can result in tearing upon release (potentially exposing the lead wires). In this configuration, we deposited the silicone/CB composite into each of the exposed channels up to approximately half their depth (using a 27-gauge needle). Then, we partially cured the composite by leaving the device in a convection oven at 60°C for 1 h.

*Step 4.* We covered the ends of copper wires (32 AWG, stranded, 0.25 mm diameter PVC insulation) with silicone/CB composite and stuck them to the partially cured composite inside each channel. These wires remained immobile due to the viscosity of the uncured silicone/CB material. Then, we fully cured the composite by leaving the device in a convection oven at 60°C for 3 h. Care should be taken to prevent the copper wires from coming into direct contact with the electrolyte, since this can generate adverse electrochemical processes. To avoid this concern at the expense of cost, platinum wire can be substituted in this step. Note that the manual assembly of wires can effectively limit the complexity of the device, as a high density of contacts (beyond four radial electrodes spanning the 5 mm circumference inside the tube) can be challenging to implement.

*Step 5.* Finally, we insulated the exposed wires and silicone/CB electrodes by drop-casting single-component silicone (RTV 3140, Dow Corning) and then carefully released the cuff from the underlying Scotch tape (assisted by immersing the device in isopropyl alcohol). Ultimately, the exposed silicone/CB electrodes were flush with the inside surface of the cuff electrode. We did not observe any residue left behind by the Scotch tape, and there were no conductivity problems associated with it. On the other hand, when we used Kapton tape (with silicone adhesive) instead of Scotch tape for the scaffold, we noticed a layer of residue on the surface of the contacts and subsequently encountered conductivity problems.

#### Composite Silicone/Carbon Electrode

We employed a composite material composed of CB and silicone—specifically, polydimethylsiloxane (PDMS)—for electrode contacts. Graphitized carbon black (Carbon black Super-P, Alfa Aesar) and Part B of Sylgard 184 (Dow Corning) were mixed 1:5 by mass using a Thinky ARE-250 planetary mixer (2000 rpm for 1 h). In the absence of a mechanical mixer, the composite can be more easily mixed by diluting it with heptane. However, all heptane should be evaporated completely before applying it to the device. Immediately prior to use, we added Sylgard 184 Part A to the composite at 8% total mass and stirred vigorously. This formulation was based on the one evaluated by Reyes et al. (2014). As an alternative, composite silicone/CB materials can be obtained commercially from manufacturers such as Creative Materials Inc.

### Design Verification

#### Electrical Characterization

The electrical characteristics of our device were evaluated using both electrochemical impedance spectroscopy (EIS) and cyclic voltammetry (CV). We performed each measurement with the VersaSTAT 3 potentiostat from Princeton Applied Research. In both modalities, our electrochemical cell comprised three electrodes immersed in room temperature phosphate buffered saline (1 X PBS, pH 7.4): one of the cuff’s cathodes as the working electrode, an Ag |AgCl| 3.0 M KCl cell as the reference electrode (grounded to enclosing Faraday cage to reduce noise), and a 1 × 2 cm^2^ section of carbon paper (Spectracarb 2050A-0550, Fuel Cell Store) as the counter electrode. According to typical EIS procedure, impedance magnitude and phase were evaluated for sinusoidal waveforms (10 mV RMS, biased to the cell’s open-circuit potential) at 60 frequencies logarithmically spaced between 1 Hz and 1 MHz. CV was performed with a scan rate of 100 mV/s between –0.7 V and 1.3 V (centered on 300 mV equilibrium potential). We chose this range based on the cathodic and anodic potentials that generated irreversible redox processes—the potentials at which the current rises precipitously.

#### Mechanical Testing

Before electrical characterization, each device was opened by 3–5 mm in a manner consistent with surgical implantation on a nerve of appropriate diameter. In addition, for a subset of our three-contact cuff electrodes, we performed extended mechanical testing. In this group, we carried out EIS and CV measurements before and after (1) the cuff was opened by 10 mm and then released, and (2) the cuff was opened until flat against a surface and then released. We repeated each mechanical operation five times successively in between measurements.

### Design Validation

The rat sciatic nerve is a commonly used *in vivo* model for testing electrodes in the PNS (Rodríguez et al., 2000; Navarro et al., 2001; Ordonez et al., 2014; Sridharan et al., 2018). This section details the execution of a motor unit recruitment in the rat sciatic nerve, which allowed us to evaluate electrophysiological characteristics such as firing threshold and spatial selectivity.

#### Surgical Preparation of Rat Sciatic Nerve and Implantation

We carried out all animal procedures under the guidelines of the Committee on Animal Care (CAC) at the Massachusetts Institute of Technology (MIT), minimizing pain experienced by the rats. Surgical exposure of the sciatic nerve followed a similar protocol to those described by Rodríguez et al. (2000), Navarro et al. (2001), Ordonez et al. (2014), and Sridharan et al. (2018).

The rat was anesthetized using a cocktail of Ketamine/Xylazine/Acepromazine (KXA; 1:0.125:0.01 ratio by mass). We administered an initial dose of 0.10 mL per 100 g of the rat’s body weight intraperitoneally through the lower right abdominal area. After one hour, successive doses at half volume were injected in 45-min intervals. We monitored the depth of anesthesia by testing the toe pinch reflex on the left hindlimb every 10 min. We positioned the rat on its abdominal side over a 37°C heating pad to maintain physiological temperature and mounted its hindlimb over a syringe to attain a more favorable exposure. The area of operation, roughly twice as large as the incision, was prepared by shaving the rat’s hair and then disinfecting the area with alternating rounds of 70% ethanol and povidone–iodine.

With the rat fixed in the prone position, we located the right femur by palpating the area and performed an incision of 3–4 cm (via #15 surgical blade) extending from the femur’s distal end towards the dorsal midline where the sciatic nerve passes through the ilium. Then, by blunt dissection with blunt hemostats and iris scissors, we separated the femoral biceps and gluteal muscles to permit access to the deep sciatic nerve. Starting 13 mm distal from the sciatic notch, we dissected the surrounding connective tissue to expose the sciatic nerve along the lateral aspect of the limb from ilium to trifurcation (approximately 2.5 cm, leaving room for slightly more than two lengths of the cuff).

After exposure, we cleaned the nerve from surrounding tissue using autoclaved cotton swabs, microsurgical anatomical tweezers, and straight scissors. We wrapped the cuff electrode around the main branch of the sciatic nerve proximal to its trifurcation, affixing sutures around the device to seal the opening. Finally, the wound was closed by suturing the skin with one layer of 5-0 Prolene surgical knots, leaving percutaneous leads for amplifier connection.

After the conclusion of the experiment, euthanasia was performed by intraperitoneal administration of KXA at double its initial dosage. After confirming the depth of anesthesia by toe-pinch, we then punctured the rat’s diaphragm, producing a bilateral pneumothorax.

#### Evaluating Muscle Responses to Cuff Electrode Stimuli

**FIGURE 3:**
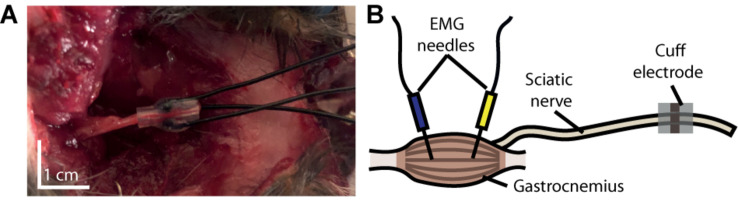
Experimental setup. **(A)** Photograph of cuff electrode implanted around the sciatic nerve of a rat following its use in an experiment. The device was inserted through an incision made on the dorsal surface of the rat’s hindlimb. **(B)** Schematic depiction of our experimental setup, illustrating the anatomical position of our cuff relative to that of the electromyographic (EMG) needles.

To demonstrate the ability of our device to activate peripheral nerve fibers and their downstream targets, we performed a motor unit recruitment study for each contact in the cuff’s cathode array. Current was driven through each electrode contact in a monopolar configuration from a current-controlled amplifier (IZ2 amplifier, Tucker–Davis Technologies). For each contact, a stainless steel electromyographic (EMG) needle inserted into the latissimus dorsi muscle (approximately 10 cm from the nerve exposure) served as the grounded return for current (see Figure 3). This experiment was executed in three phases, each consisting of a ramp of pulses applied at one of the three contacts. Each ramp consisted of 20 steps spaced evenly between 10 μA and 200 μA, and each step consisted of three 1 ms monophasic pulses triggered 3 s apart.

Compound muscle action potentials (CMAP), which proxy upstream activity of innervating fibers, were recorded from pairs of EMG needles inserted with a gap of 1 cm into the gastrocnemius, tibialis anterior, and biceps femoris muscles. With a differential amplifier (PZ2 amplifier, Tucker–Davis Technologies), we acquired each channel versus a distant reference needle inserted into the latissimus dorsi muscle opposite to IZ2 ground (sampled at 25 kHz).

For each pulse driven through the cuff electrode, we quantified the downstream activation of each muscle by the peak rectified amplitude of the elicited CMAP. We constructed recruitment curves by averaging the triplicate pulses from each step and plotting them versus the stimulus amplitude at that step. All post-processing was performed in Matlab from Mathworks and the Julia programming language.

## Results

### Labor, Cost, and Success Rate

As demonstrated in the materials and methods section, the fabrication process described herein incorporated only inexpensive materials that could be purchased from mainstream commercial suppliers. We determined that each batch of four devices cost approximately $8.80 of materials. Ultimately, our process required no specialized fabrication experience and minimal labor hours: three to four hours for one person to produce a batch of four to five devices. In addition to labor, each batch requires four hours of unsupervised curing time in the middle of the process, and it requires the device to cure overnight in the final step. The silicone/CB composite itself required two hours to produce; however, it can easily be produced in sufficient quantity to serve many batches. Finally, each new electrode configuration required approximately one to two hours to implement in CAD, and an extra one to two hours during laser cutting to verify the pattern and optimize cutting parameters. In contrast, Foldes et al. (2011) report that their process for manufacturing cuff electrodes with longitudinally arranged contacts calls for eight hours of labor.

Based on visual inspection and electrical characterization (see following section), we determined that our fabrication process produced consistent, properly functioning devices. As shown in Supplementary Figure 1, this process successfully rendered silicone/CB contacts with dimensions of 200 μm. The silicone tubing, silicone/CB composite, and RTV 3140 all adhered durably to each other. We observed defects in only two out of fifteen cuff electrodes. The mode of failure in both of these devices was tearing in the silicone/CB electrodes, which short-circuited the lead wire to the electrolyte solution. These defects were inferred from visual imperfections in the material, lower transfer resistance measured by EIS, and abnormal redox peaks measured by CV (see Supplementary Figure 2).

Tearing is a consequence of the reduced flexibility of the silicone/CB composite versus unmodified silicone (Eklund and Kjäll, 2019). Since only two of fifteen devices failed by this mechanism, we conclude that the mechanical difference between silicone and CB had a minimal impact on the 0.9 × 1.4 mm^2^ contacts. We did not test electrodes with larger contacts extensively, but we found that 0.9 × 6 mm^2^ contacts would frequently tear (see Supplementary Figure 1). This tearing would occur during the curing process, likely due to the accompanying change in volume. Therefore, large-area patterns will require more careful design.

In order to better understand the mechanical limitations of these devices, we subjected two of our three-contact cuff electrodes to the mechanical testing procedure described in the “Mechanical Testing” section. Of the six electrode contacts we tested, we found that defects were introduced in only one case after repeatedly flattening the cuff against a surface. CV measurements indicated that this contact became torn, shorting the lead wire to the electrolyte (see the “Electrochemical Characteristics” section). Of the remaining contacts, the relative change in electrode impedances, (1) before and after opening by 10 mm was −12.1 ± 6.4 % (*n* = 6), and (2) before and after flattening was −22.1 ± 9.4 % (*n* = 5). For the CV profiles accompanying one of these contacts, see Supplementary Figure 3. This shift suggests that some structural changes developed during the mechanical tests that exposed a larger surface area of the electrode. As these structural changes may introduce some variability into the device’s performance, effort should be taken to prevent extensive stretching or compression. On the other hand, this consequence is relatively minor compared with the constriction and piercing of nerves highlighted by Haugland (1996) for cuffs with hand-sewn wires.

### Electrochemical Characteristics

**FIGURE 4:**
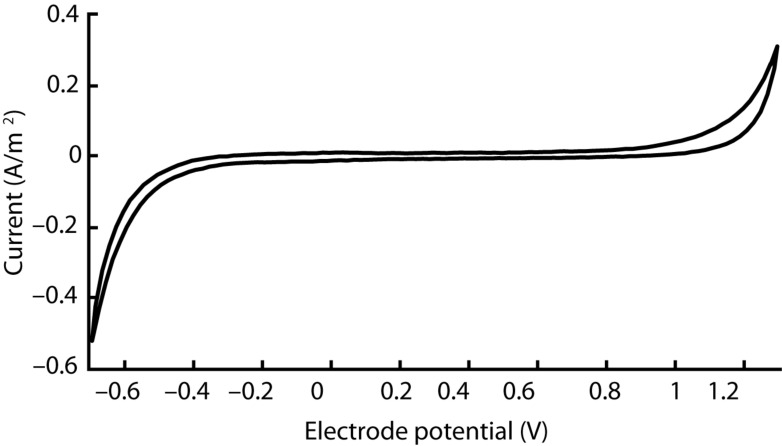
Cyclic voltammogram acquired for a single silicone/CB composite electrode scanned at 100 mV/s between −0.7 V and 1.3 V. This outcome is representative of four other electrodes that we evaluated in our study.

The cyclic voltammogram of our silicone/CB contact, as shown in Figure 4, is essentially featureless, suggesting the absence of any significant Faradaic charge transfer. Such characteristics, typically attributed to a double-layer charging/discharging mechanism, are ideal for biological applications. Based on the sharp increases in current, we judged that electrolysis begins around –0.6 V and 1.2 V for the cathode and anode respectively. We observed no gas bubbles emanating from the contact during the course of polarization, further indicating that safe window for polarization of this material occurs from –0.6 V to 1.2 V.

Meanwhile, a distinctly different CV profile could be observed in defective cuffs. In cases where the electrode was damaged, leaving part of the lead wire exposed, the reduction and oxidation peaks shifted to –0.3 V and 0.8 V respectively (see Supplementary Figure 2). Additionally, the impedance became substantially lower. Thus, defective devices could be discriminated clearly from their electrochemical characteristics.

**FIGURE 5:**
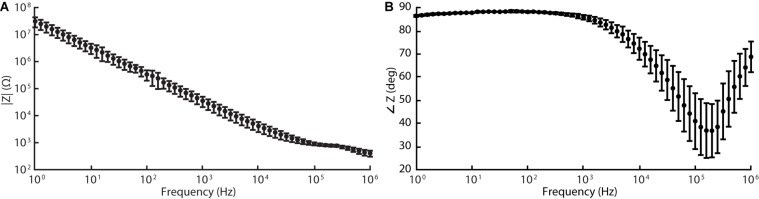
Electrochemical impedance spectroscopy (EIS) data acquired for silicone/CB composite electrode at 60 frequencies logarithmically spaced between 1 Hz and 1 MHz (10 mV RMS, biased to the cell’s open-circuit potential). Graphs **(A)** and **(B)** represent the magnitude and phase components of the Bode plot respectively.

The silicone/CB composite electrode exhibited a larger impedance than conventional electrode materials such as platinum (see Table 2 for a comparison). Our EIS measurements revealed the impedances of the tested electrode contacts as 36 ± 15 kΩ (*n* = 5) for 1 kHz sinusoidal polarizations (see Figure 5 for full spectrum). As is the case for several of the items in Table 2, the larger impedance of silicone/CB arises from the insulating nature of silicone.

**TABLE 2:** Comparison of areal impedances for several electrode materials (in 1 X PBS, pH 7.4, at room temperature) reported in the literature (each having original dimensions on the scale of ∼1 mm).

Material	|Z| (kΩ mm^2^) at 1 kHz	References
PDMS/CB (this study)	45.4 (*n* = 5)	–
Platinum foil	0.317 (*n* = 10)	Lee et al., 2010
PDMS/CNTs	43.5 (*n* = 3)*	Behrens et al., 2018
PDMS/Pt powder	1.05 (*n* = 8)	Schiavone et al., 2018

*According to the same reference, impedance can be reduced to 3.2 kΩ mm^2^ by dry etching with CF_4_/O_2_ gas.

Regardless, the impedances of our electrodes were sufficiently low that monophasic pulses, 200 μA amplitude and 1 ms pulse width (according to parameters implemented during *in vivo* stimulation described in the following section), maintained the electrode potential within the safe window of polarization defined above. Thus, during its intended operation as a peripheral nerve stimulator, our cuff electrode will function safely within the limits of (1) the electrode’s electrochemical window, and (2) typical stimulation amplifiers.

### Recruitment of Motor Units

The surgical implantation of the cuff took approximately 10 min and did not require any specialized instruments (see Figure 3A). Based on visual inspection, the device remained compliantly seated throughout the experiment inside the cleft between the femoral biceps and gluteal muscles without any external supports. In addition, its format allowed the skin to be re-sutured shut over the incision after implantation, limiting desiccation and hypothermia of the surgical site. Upon removal of the cuff, we observed no morphological aberrations of the nerve along the location of cuff attachment.

**FIGURE 6:**
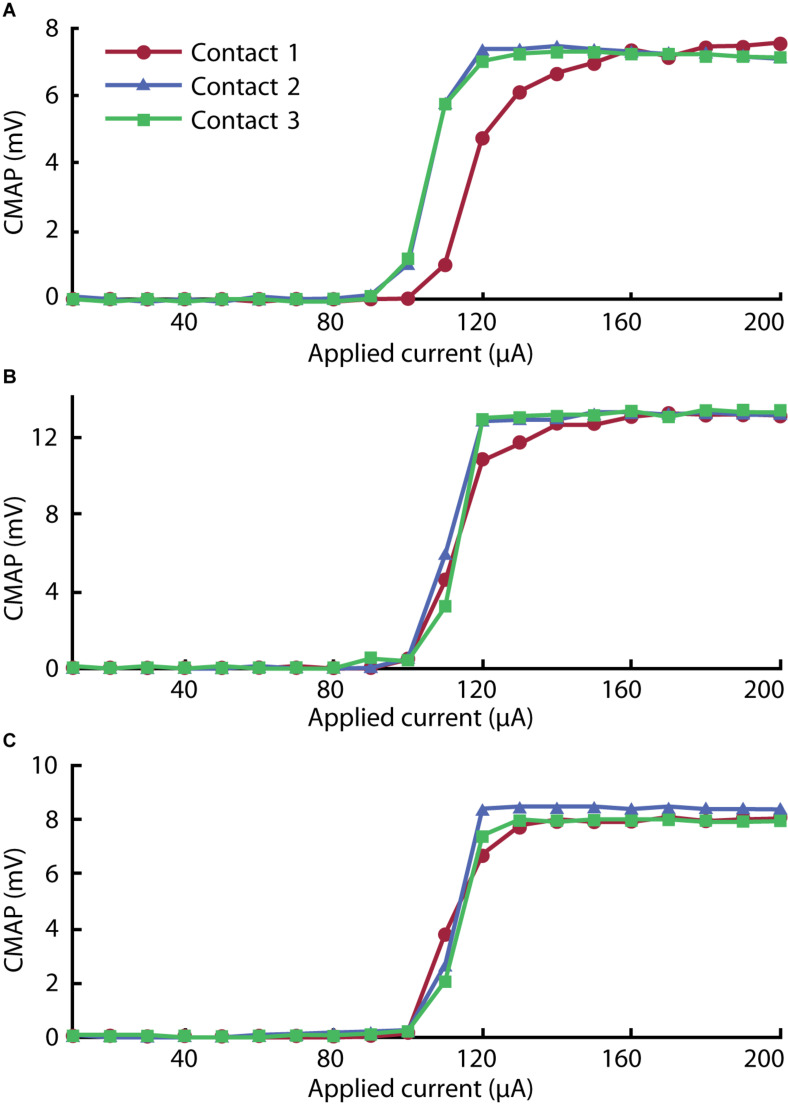
Recruitment curves for **(A)** gastrocnemius, **(B)** tibialis anterior, and **(C)** biceps femoris muscles stimulated with each of the three contacts on the cuff electrode. The peak rectified compound muscle action potential (CMAP) acquired for three pulses was averaged in each step to give each value across the range of the ramp sequence.

After successful implantation, we executed a motor unit recruitment study to verify activation behavior characteristic of peripheral nerve fibers. As shown in Figure 6, we observed robust muscle activity in response to excitatory stimulus (for raw traces, see Supplementary Figure 4). The magnitude of elicited CMAPs increased monotonically with stimulus amplitude in a distinctly sigmoidal manner. The thresholds (quantified by the stimulus amplitude at half maximum) corresponding to each curve were consistent with typical cuff electrodes reported in the literature (Rodríguez et al., 2000).

In the gastrocnemius channel, plotted in Figure 6A, we observed lower activation thresholds associated with Contacts 2 and 3 than with Contact 1. We attribute this to the selective influence of contacts on adjacent regions of nerve. As reported by Navarro et al. (2005), selectivity can be improved by incorporating more contacts into the array.

## Discussion

As outlined above, this process requires negligible costs relative to commercial solutions. At the same time, compared to other facile manufacturing processes reported in the literature, it requires fewer labor hours. However, its key advantage is that, as a facile method, it can produce devices with precise, intricate electrode geometries. The precision is limited only by the dimensions of the laser’s etch, which, for the PLS4.75 from Universal Laser Systems, was ∼200 μm. On the other hand, we found that the manual assembly of wires in Step 4 could present a challenge for designs with densities of contacts greater than ∼1 contact/mm.

Although contacts as small as 0.2 mm can be embedded in the cuff, based on our experience with this process, we expect that its principles will be most viable for cuffs with inner diameters of at least 0.5 mm. Thus, future investigators can use this report as a basis for constructing cuff electrodes for smaller nerves, such as the rat vagus nerve whose diameter Pelot et al. (2020) report is 0.260 ± 0.025 mm at the cervical level (Noller et al., 2019).

While this work focused on devices for acute experiments, one could readily adapt it to long-term implants. Considering the tighter constraints on biocompatibility and stability, devices will need to be constructed with platinum lead wires with Teflon insulation, medical grade silicone tubing for the substrate (available from suppliers such as Trelleborg), and medical grade PDMS for the electrode composite and the device’s encapsulation. Furthermore, the lead wires should be made as thin as possible in order to improve mechanical compatibility. Regarding the electrode material itself, silicone/CNTs and silicone/graphite composites have identical biocompatibility to that of silicone given a sufficiently low content of the conductive element (Blau et al., 2011; Kim et al., 2018). Silicone/CB likely has similar biological inertness to these other carbon-based composites, but this tangential evidence will need to be supported by future investigation in order to verify suitability for long-term implantation.

This method fundamentally relies on the use of a conductive silicone composite material for electrode contacts—other materials would fail to integrate with the body of the cuff. Although we considered several variations of this material based on prior literature, each potentially having their own advantages, we chose silicone/CB due to favorable cost, ease of fabrication, and availability of a commercial product. However, its specific role as a neural stimulating electrode is relatively nascent, and, thus, it currently lacks extensive electrochemical and biological characterization. We deemed the silicone/CB material suitable for our particular application by subjecting it to appropriate electrochemical testing. At this point, we recommend that new designs, likewise, undergo verification by EIS and CV. As discussed in see the “Electrochemical Characteristics” section one can clearly identify defective devices from CV and impedance measurements, the latter being available *in situ*.

The primary limitation of silicone/CB that we have determined from the results described here is its impedance—as demonstrated from EIS, this material has a much larger impedance than conventional electrode materials like platinum. This may limit performance in applications where smaller contact sizes and larger currents are necessary. In addition, it limits the application of this material to stimulation, as recording requires low-impedance electrodes. While increasing the content of CB is untenable due to its negative effect on mechanical strength, improvements could be made by substituting silicone/CB with another compatible silicone composite such as silicone/CNTs or silicone/platinum. Alternatively, hybrid composites such as CB with silver particles could be considered, which demonstrate improved conductivity relative to silicone/CB on its own (Eklund and Kjäll, 2019). These modifications have the potential to enable rapid manufacturing of cuff electrodes suitable for electrophysiological recordings. Furthermore, these alternate materials may allow the total content of conductive material to be reduced, improving mechanical properties and permitting larger or more durable electrode contacts.

## Conclusion

No specialized equipment or fabrication experience was required in our production, and we encountered negligible costs relative to commercially available solutions. Based on our characterization, which included electrochemical and mechanical testing, we can confirm that the circumpolar cuffs generated under our process were consistent and effective. In addition, our limited electrophysiological study suggests selective activation of the gastrocnemius muscle by individual contacts. This outcome was enabled by precise tool-aided patterning and the use of flexible silicone-based composite electrodes. Not relying significantly on fine motor or other specialized skills, this process offers a distinct advantage over other facile methods, such as those requiring hand-stitching and manual patterning of metal films. Furthermore, silicone/CB cuff electrodes are comprised exclusively of soft materials (with the exception of lead wires), removing the risk of nerve piercing or constriction.

At this stage in its development, we do not expect that this method will displace conventional lithographic techniques for most final-stage productions. However, its qualities make it well suited for rapid manufacturing of peripheral neuro-modulation schemes. In this capacity, its use cases include: (1) manufacturing cuffs electrodes in cases of low, sporadic demand, (2) design-stage production for quick iteration of ideas, and (3) manufacturing customizable products. With the expansion of commercial neuro-technology, we anticipate a larger demand for technologies that can address these production requirements.

Considering the significant variation in both PNS anatomy and the applications of FES and neuro-modulation modalities, a personalized approach could greatly improve patient outcomes. The rapid manufacturing process we present moves us closer to this goal for cuff electrode devices.

## Data Availability Statement

The raw data supporting the conclusions of this article will be made available by the authors, without undue reservation.

## Ethics Statement

The animal study was reviewed and approved by Institutional Animal Care and Use Committee at the Massachusetts Institute of Technology (protocol no. 0220-011-23).

## Author Contributions

MF performed conceptualization, methodology, software, validation, formal analysis, investigation, and writing—original draft. MP and SA performed methodology, investigation, and writing—review and editing. AL performed methodology, software, investigation, resources, and writing—review and editing. CL and RA performed methodology, writing—review and editing, and supervision. SL performed writing—review and editing, supervision, project administration, and funding acquisition. JH performed conceptualization, writing—review and editing, supervision, project administration, and funding acquisition. All authors contributed to the article and approved the submitted version.

## Conflict of Interest

The authors declare that the research was conducted in the absence of any commercial or financial relationships that could be construed as a potential conflict of interest.
