# Supplementary material for: Rapid and Low Cost Manufacturing of Cuff Electrodes
Source: Front Neurosci. 2021 Feb 16;15:628778. doi: 10.3389/fnins.2021.628778 (PMC7920973; doi:10.3389/fnins.2021.628778)
Supplement: Supplementary file 1 [file Image_1.PDF]

Supplementary Material

Rapid and low cost manufacturing of cuff electrodes

Matthew T. Flavin,[1,2] Marek A. Paul,[3,4] Alexander S. Lim,[5] Senan Abdulhamed,[3] Charles A. Lissandrello,[2,*] Robert Ajemian,[5] Samuel J. Lin,[3] Jongyoon Han[1,*]

[1]Department of Electrical Engineering & Computer Science, Massachusetts Institute of Technology, Cambridge, MA 02139, USA

[2]Bioengineering Division, Draper, 555 Technology Square, Cambridge, MA 02139, USA

[3]Division of Plastic and Reconstructive Surgery, Department of Surgery, Beth Israel Deaconess Medical Center, Harvard Medical School, Boston, MA 02215

[4]Department of Plastic Surgery, Lower Silesia Specialist Hospital, 54-049 Wroclaw, Poland

[5]McGovern Institute for Brain Research, Massachusetts Institute of Technology, Cambridge, MA 02139, USA

*** Correspondence:**
Jongyoon Han
jyhan@mit.edu

Charles Lissandrello
clissandrello@draper.com

1 Image of cuff electrode contacts

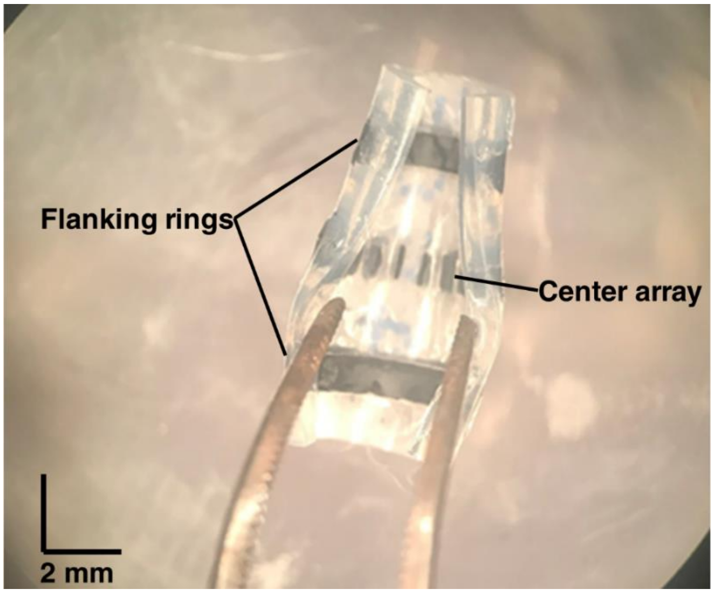

Supplementary Figure 1. Image of the inside surface of a circumpolar cuff electrode with eight radially arrayed contacts and two flanking rings, excluding attachment of wires. Each of the center contacts are 1×0.2 mm^2 and extend 0.8 mm into the body of the cuff (distorting the boundary of the electrodes in this image). This image demonstrates slight tearing in each of the flanking electrodes (discussed in Section 3.1).

2 Cyclic voltammetry profile of defective electrode contacts

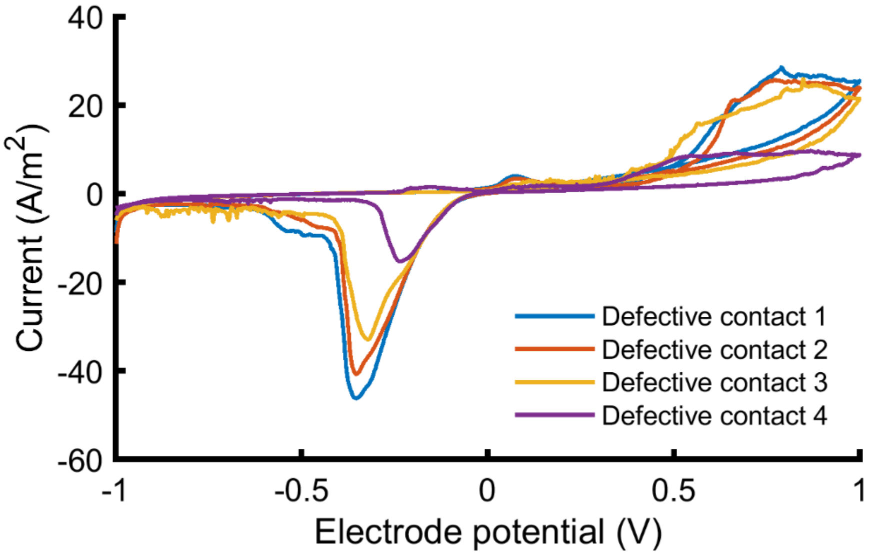

Supplementary Figure 2. Cyclic voltammogram acquired for several defective silicone/CB composite electrode, scanned at 100 mV/s between –1 V and 1 V (single-cycle).

3 Cyclic voltammetry profile during mechanical testing

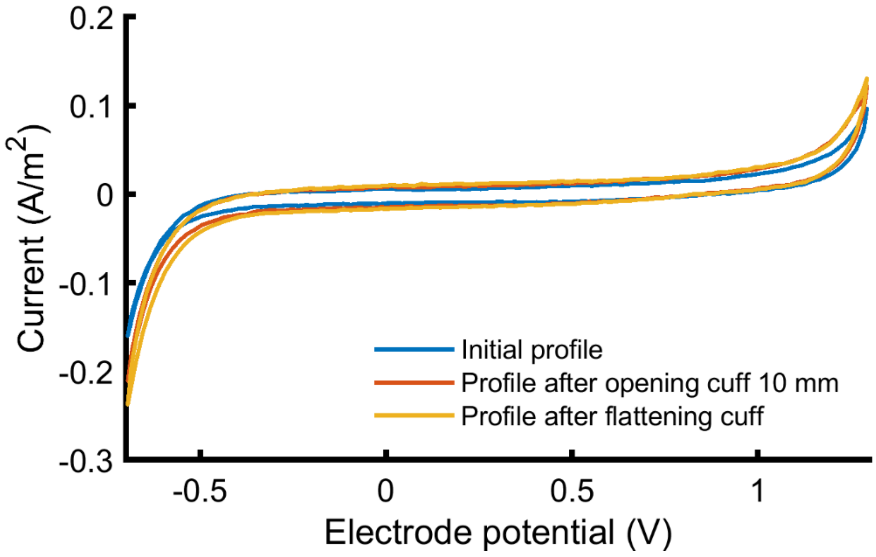

Supplementary Figure 3. Cyclic voltammograms acquired for a single silicone/CB composite electrode during the mechanical testing procedure described in Section 2.2.2, scanned at 100 mV/s between –0.7 V and 1.3 V (last of ten cycles).

4 Raw data from electrophysiological measurements

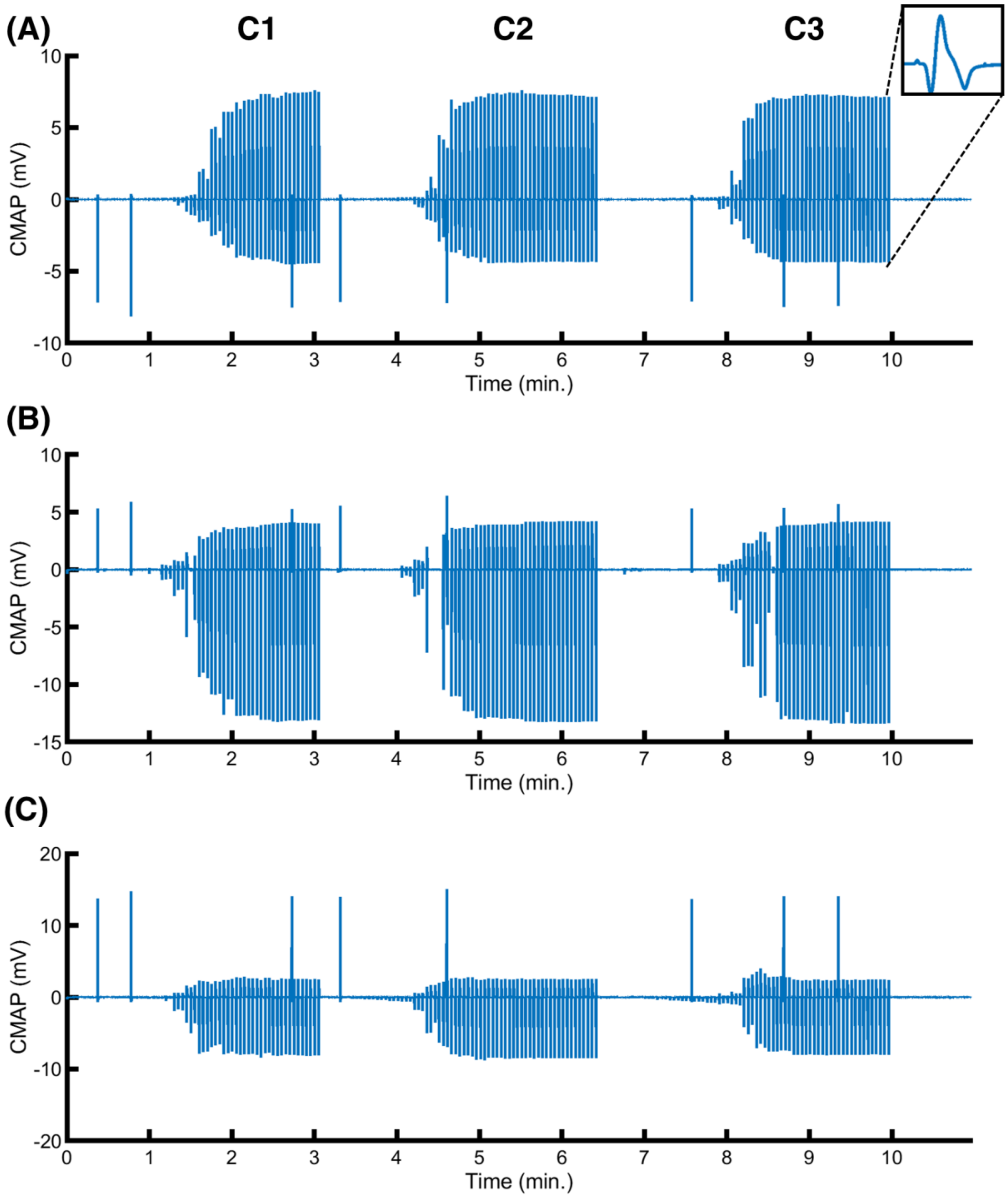

Supplementary Figure 4. Raw data for electrophysiological measurements performed during cuff electrode stimulation. This electrode had three contacts arrayed radially on the inside of the cuff. Current was driven through each electrode in a monopolar configuration, returned in both cases through a distant ground needle inserted into the rat's shoulder muscle. This experiment was executed in three phases: (I) ramp on Contact 1 (C1), (II) ramp on Contact 2 (C2), (III) ramp on Contact 3 (C3). Each ramp consisted of 20 steps spaced evenly between 10 µA and 200 µA. Three monophasic pulses were applied during each step with pulse–widths of 1 ms and inter-pulse periods of 3 s. Compound muscle action potentials (CMAPs) were acquired from intramuscular electromyographic (EMG) needles inserted into the **(A)** gastrocnemius, **(B)** tibialis anterior, and **(C)** biceps femoris muscles. The inlet shows an isolated CMAP from the gastrocnemius channel.
